# PhytobezoarInduced Small Bowel Obstruction in a Young Male with Virgin Abdomen

**DOI:** 10.4172/2161-069X.1000266

**Published:** 2015-03-25

**Authors:** Edward P. Manning, Vikram Vattipallly, Masooma Niazi, Ajay Shah

**Affiliations:** 1 Department of Medicine, Hospital of the University of Pennsylvania, Philadelphia, PA, USA; 2 Department of Surgery, Bronx Lebanon Hospital, Bronx, NY, USA; 3 Department of Pathology, Bronx Lebanon Hospital, Bronx, NY, USA

**Keywords:** Phytobezoar, Small bowel obstruction, Diabetes, Opiate abuse, Acute abdomen

## Abstract

Phytobezoars are a rare cause of small bowel obstruction. Such cases are most commonly associated with previous abdominal surgery or poor dentition or psychiatric conditions. A 40 year old man with a virgin abdomen and excellent dentition and no underlying psychiatric condition presented with an acute abdomen. CT scan revealed a transition point between dilated proximal loops of small bowel and collapsed distal loops. Exploratory laparotomy revealed a phytobezoar unable to be milked into the cecum and an enterectomy with primary anastamosis was performed without complication. A detailed history revealing several less common predisposing factors for phytobezoars should increase clinical suspicion of a phytobezoarinduced small bowel obstruction in the setting of an acute abdomen. Vigilance in presentations of an acute abdomen improves the usefulness of medical imaging, such as a CT, to detect phytobezoars. Understanding mechanisms of phytobezoar formation helps guide management and may prevent surgery.

## Case Presentation

A 40 yearold man presented to the Emergency Department (ED) with a twoday history of abdominal pain. He described the pain as insidious in onset, initially diffuse and later localized to the umbilical region, nine out of ten intensity. His pain was associated with nausea and vomiting. His last bowel movement was one day prior to presentation, but he was passing flatus in the ED. His past medical history was significant for diabetes, hypertension, and hepatitis C. He was diagnosed with diabetes approximately two months prior to admission after complaining to his primary care physician of blurry vision of a few months duration. His hypertension was being controlled with diet and lifestyle changes, including eating a healthy diet. The patient denied any surgeries in the past. His social history was significant for a fouryear history of intravenous heroin abuse as well as occasional marijuana use. His last use of heroin was five months prior to presentation. He has been in a substance abuse treatment program for the past five months but his treatment has not included the use of methadone. His home medications included metformin for diabetes.

On physical exam, the patient was alert and in acute distress due to abdominal pain. Vitals were stable: afebrile, blood pressure 134/86, respiratory rate of 16 breaths per minutes, oxygen saturation of 99% on room air. Pupils were equal round and reactive with anicteric sclera. Dentition was good, mucous membranes moist, oropharynx without lesions and nonerythematous. Cardiovascular exam revealed regular rate and rhythm, S1 and S2 sounds auscultated without rubs, murmurs, or gallops. Respiratory exam was unremarkable with normal findings on percussion, clear to auscultation bilaterally without adventitious sounds. His abdomen was soft, mildly distended with tenderness and guarding in the umbilical region. There was no rebound tenderness and no rigidity. No scars were visible on the abdomen, and his rectal exam was normal. No rashes were observed, and the patient was neurologically intact, alert, oriented, and responding appropriately without focal deficits.

**Figure 1:**
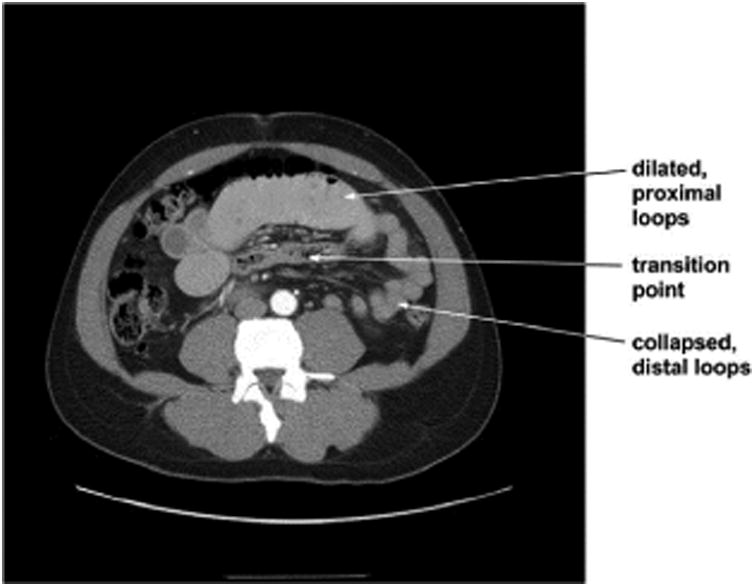
CT of patient revealing dilated loops of small bowel (proximal) and collapsed loops of small bowel (distal).

Routine labs were all within normal limits except for mild leucocytosis. Laboratory examination at that time revealed a HgbA1C level of 9.9%. Urine toxicology screening was positive for canniboid. A contrastenhanced CT was obtained, as shown in Figure 1, which revealed dilatation of proximal small bowel loops associated with a transition point and collapsed, distal loops of small bowel.

The surgical team assessed the patient to be a young man with an acute, virgin abdomen with transition point requiring exploratory laparotomy. Differential diagnosis included a neoplasm or congenital band. The patient was prepared for surgery.

## Introduction

Phytobezoars are undigested vegetable in the gastrointestinal system and a rare cause of small bowel obstruction [1,2] typically associated with prior abdominal surgery and poor dentition [3-5]. Preoperative diagnosis of a phytobezoar is not common, but when successfully diagnosed effective medical management is possible [6]. In the setting of an acute abdomen, surgery is necessary to remove the phytobezoar and ensure it does not recur. Here we present a unique case of small bowel obstruction secondary to phytobezoar in a young patient with a virgin abdomen and excellent dentition. We review the literature on predisposing factors, discuss the pathophysiology of phytobezoars, and how this relates to management options. Finally, we draft a current comprehensive algorithm regarding management of phytobezoars.

A phytobezoar is undigested vegetable matter found in the digestive system (stomach, small intestine, or colon) that causes obstruction, often composed of indigestible cellulose, tannin, or lignin derived from ingested vegetables and fruits [1,2]. The word “bezoar” comes from the Farsi word “ (padzahr)”, which means “antidote” or “antitoxin” [7]. This stems from the belief that bezoars were the universal antidote to poisons; therefore, they have been historically valued amongst nobility for these alleged properties [8,9]. It has been reported that a trichobezoar (bezoars formed from hair) immersed in an arseniclaced solution can remove the toxic metabolite arsenite by binding it to sulfur compounds found in the hair [9]. Anectdotal evidence also exists that describes failed attempts of using bezoars to protect humans from the effects of poison [8]. Further, animal bezoars (such as ox bezoars or gallstones) are used in Eastern medicine in the effort to remove toxins from the body.

Other types of bezoars include: [1,2,10]

trichobezoar: hair bezoar, associated with trichotillomania and other psychologic disorders

pharmacobezoars: medicine/pill casing bezoar, associated with overdoses

diospyrobezoar: persimmon bezoar, associated with ingestion of unripe persimmons

lactobezoar: milk bezoar, associated with inspissated milk in premies.

Phytobezoars are an uncommon cause of small bowel obstruction with an incidence of approximately 1.5%, [3,11] accounting for as many as 4% of small bowel obstructions [12]. The leading predisposing factors for small bowel obstruction due to phytobezoars are: previous gastric surgery, previous abdominal surgery, and the absence of teeth [3]. In a study of 87 cases of intestinal bezoar, 76.3% had a history of previous abdominal surgery the majority of which were bilateral truncal vagotomy plus pyloroplasty, excess ingestion of vegetable fiber in 39.5% of the cases, and substantial changes in dentition in 24% of the cases [4,5]. Phytobezoars have also been associated with intestinal diverticula, tumors, and even Chow Mein noodles [13]. While rare, bezoars are the most common foreign body found in the gastrointestinal tract. The most common form of bezoar is a phytobezoar [2]. Certain fruits, such as unripe persimmons, pineapples and prickly pears (cactus fruit/figs), are associated with bezoar formation [2,12,14].

The pathophysiology of bezoar formation can be broken into mechanical or chemical components, as characterized in Table 1. This helps explain the effectiveness of certain methods of management. For example, a function of the pylorus is to prevent poorly hydrolyzed and mechanically fragmented boluses of food from passing into the small bowel.

Dysfunction or elimination of pyloric function as the result of Bilroth I or II procedures allows poorly hydrolyzed food matter into the small bowel which increases production of phytobezoar formation [6,15]. Gastrointestinal immotility, such as gastric stasis or delayed emptying, prolongs retention of material in the intestines, thus promoting the formation of bezoars. [12,16]. The increased transit time may also increase the probability of food impaction. Primary small bowel bezoars are very rare and usually form secondary to underlying small bowel disease such as a diverticulum, stricture or tumor. Therefore, such cases are commonly associated with previous surgery, Crohn's disease, congenital diverticula or tuberculosis [4,17-22].

Hypoacidity, such as in the case of truncal vagotomy, decreases hydrolysis of ingested food thus increasing the amount of undigested food matter passed into the small bowel [16]. Increased intake of indigestible matter, such as hair (trichobezoar), or poorly masticated food has a similar effect. This increases the probability of bezoar formation, particularly phytobezoars.

This also increases the probability of obstruction of the terminal ileum, since the terminal ileum is the narrowest portion of the small bowel. Finally, interactions between ingested matter and the gastrointestinal environment may result in rather unusual cases of bezoars, such as the ingestion of unripened persimmons (diospyrobezoar) or inspissated milk in underdeveloped gastrointestinal systems (lactobezoar). For example, in the case of diospyrobezoars, the ingestion of unripened persimmons introduces a high concentration of tannin and shibuol into the highly acidic environment of the stomach. Gastric acid polymerizes these substances thus providing a nidus for bezoar formation [15,23].

Phytobezoars have no unique signs or symptoms associated with their presentation. Most intestinal bezoars present as a complete bowel instruction. (Escamilla 1994) Small bowel obstructions due to phytobezoars often present with crampy abdominal pain with vomiting. While prior surgery is a predisposing factor for phytobezoars, it is not a necessary condition [13]. Highpitched bowel sounds may or may not be present though in the absence of adhesions due to a prior surgery, Strictures due to an underlying conditions such as Crohns, a diuretic, or tumor is usually found [13]. A more recent study of 15 cases of small bowel obstruction due to phytobezoars in 2008 showed that 13 of the 15 cases presented free of fever with no peritonitis though nasogastric drainage revealed bilious fluid. Only two cases presented as acute abdomens requiring emergency surgery [12].

Because they are so rare, phytobezoars are often difficult to recognize and diagnose. Phytobezoars should be suspected in patients who have had previous gastric or abdominal surgery, extremely poor dentition, or sufficiently increased fiber intake prior to presentation [3]. While physical examination, abdominal Xray, and small bowel study have been traditionally recommended in suspected cases of phytobezoars, the sensitivity of such diagnostic studies is only 10% [12]. Barium studies reveal an intraluminal filling defect, suggesting a mass not fixed to bowel wall with a mottled appearance similar to a Wilms tumor [15]. On ultrasound, a phytobezoar appears like a hyperechoic surface with acoustic shadowing [15].

CT remains the best modality to diagnose a phytobezoar; however, it can often be misdiagnosed in favor of more common sources of bowel obstruction such as intussusception. CT has a positive predictive value of only 20%, [12,24] revealing a stoollike mass with a solid rim and heterogenous, “mottledgas” appearing center unable to take up iodine contrast in between proximal, distended loops of bowel and a distal, collapsed loops of bowel [12,13].

On laparotomy, a distended ileum with a mobile intraluminal mass near the ileocecal valve, the narrowest portion of the small bowel, is often found [13]. The mass is generally followed by collapsed bowel. The small bowel is often not ischemic unless it is found very late in the presentation. A thorough examination of the intestines and stomach is necessary during surgery, as concomitant bezoars is not uncommon [6].

The literature shows that phytobezoars have been managed in numerous surgical and medical manners that share a favorable prognosis. In a study of 87 cases of phytobezoars, in which all cases were treated surgically, digital fragmentation and milking of the bezoar into the cecum were initially attempted. Enterotomy and bezoar extraction were subsequently performed if fragmentation and milking of the bezoar were not possible [5].

In a later retrospective study of 375 patient hospitalized for small-bowel obstruction, 15 cases (4%) were secondary to phytobezoars. Of these 15 cases, diagnosis was made by CT in three patients and obstruction was subsequently relieved in these three cases with gastric aspiration, avoiding surgery. The remaining twelve underwent laparotomy during which the bezoar was fragmented digitally and washed into the colon [12].

Laparoscopic treatment of bezoars has also been described as an effective means of treating bezoars in the small intestine [25]. The evidence suggests that if a phytobezoar is diagnosed prior to surgery, it is likely that medical treatments will be successful.

Numerous medical therapies have been attempted with success in clearing phytobezoars, including Coca Cola, [26-28], Adolph's Meat Tenderizer, [29] Lcysteine, cellulase, [30,31] cellulase with metoclopramide, [32] papain, [31] water jet, pineapple juice, normal saline, 0.1 M hydrochloric acid, sodium bicarbonate, pancrealipase, pancreatin, and 12% zinc chloride [2].

These therapies are not without complications and should be reviewed in detail before attempts at using them [2,31]. While some of these medical treatments may seem bizarre or anectdotal in nature, some of them, such as a CocaCola lavage, have recently been found to be effective based on systematic review (greater than 50% effectiveness when used as a stand alone therapy and greater than 90% as an adjuvant to laparoscopic techniques) [33].

In reviewing the literature, the goal of treatment should be to clear the intestinal lumen of the phytobezoar and prevent recurrence [34]. Primary attempts toward treatment of phytobezoars in the small intestine in stable patients should focus on medical treatment.

Secondary treatment should involve milking of the phytobezoar into the cecum. An enterotomy is indicated if the bezoar cannot be fragmented and milked into the cecum. Finally, a resection is indicated in cases of intestinal necrosis, failure of the bezoar separating from the intestinal mucosa, and anticipated recurrence of phytobezoar.

**Figure 2:**
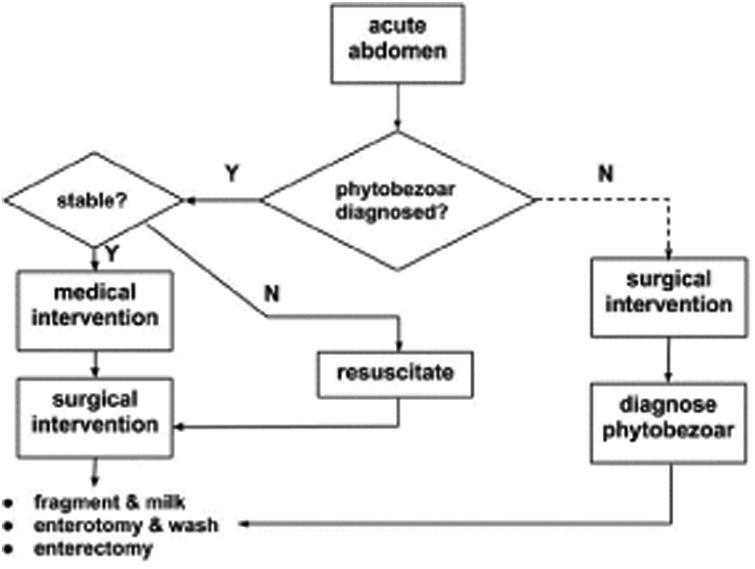
Algorithm of phytobezoar management in patients presenting with abdominal pain based on a review of current literature.

Surgery, when warranted, should not be delayed as it is associated with prolonged postoperative hospitalization [12,35]. A review of the management of phytobezoars is summarized in Figure 2.

## Case Continuation and Discussion

**Figure 3:**
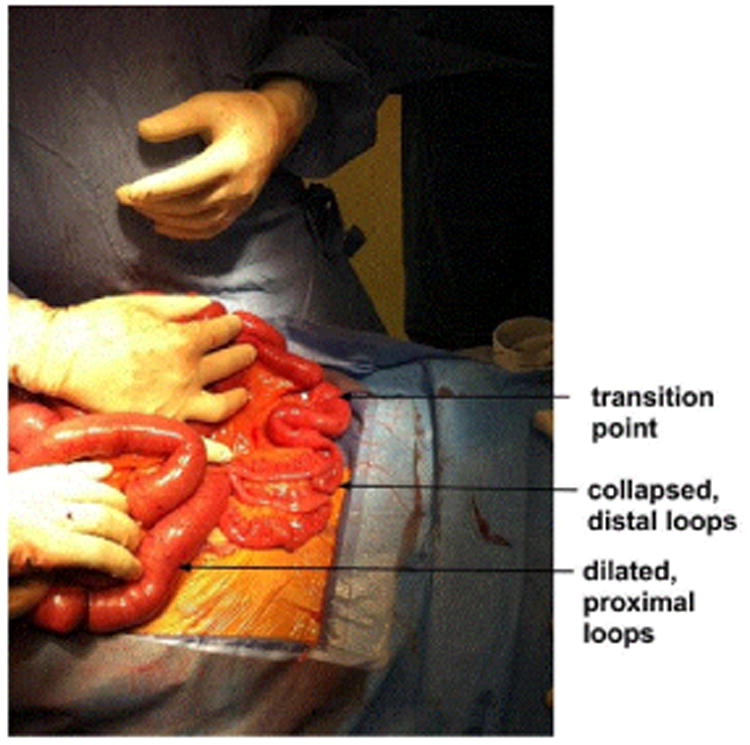
The removed specimen opened at the back bench revealed a phytobezoar approximately 4 by 5 cm. Remnants of peas, string beans, and carrots are present. Pathology revealed no polyps, tumors, or strictures but did reveal the diameter of the specimen of distal ileum to be approximately 4 cm. This places the diameter of this patient's distal ileum below the 5th percentile of diameters of the distal ileum, the average of which is 18.9 mm (S.D. 4.2 mm) [37]. Reference bar = 22 cm.

Exploratory laparotomy was performed, revealing dilated proximal small bowel loops with some congestion, collapsed distal small bowel loops, and a transition point in the midileum approximately 45 cm from the ileocecal junction, as shown in Figure 3.

The transition point contained thick, viscous material and a suspicious polypoid mass with a likely tree top mobility distally. An attempt was made to digitally fragment the material and milk it toward the cecum; however, the mass was fairly immobile.

**Figure 4:**
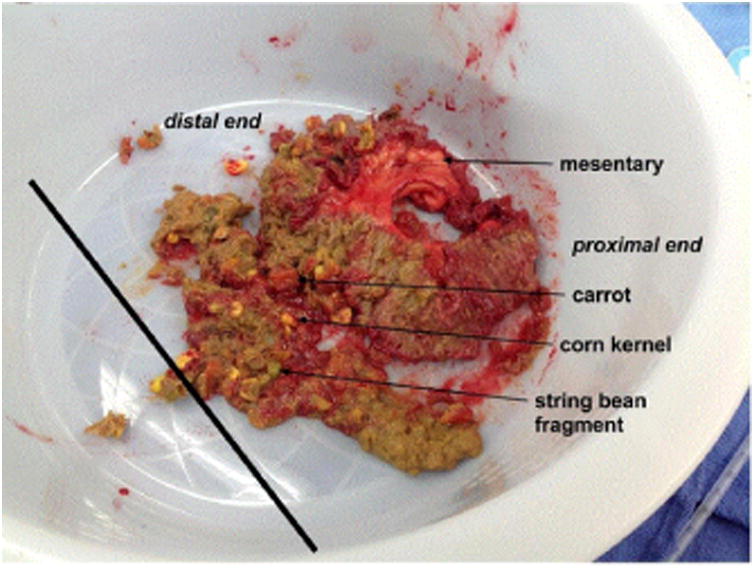
Intraoperative photograph reveals the transition point separating proximal, dilated loops of small bowel and distant, collapsed loops of small bowel. The thick material in the transition point was unable to be digitally fragmented or milked into the cecum. A polypoid mass, later found to be a corn kernel, in the distal portion of the transition point concerned the surgical team, leading to an enterectomy of approximately 30 cm in length.

An enterotomy was performed, revealing fragments of vegetable matter. Approximately 30 cm of suspicious pathological small bowel was resected and anastomosed. The isolated small bowel specimen was opened on the back bench, as shown in Figure 4, revealing a bulky, yellowtan pasty material with vegetable fragments including corn kernels and green vegetables such as peas and string beans.

Pathology confirmed an intraluminal phytobezoar (approximately 5 cm × 4.5 cm) adherent to the mucosal surface of the specimen. Adjacent mucosa were dilated and inflamed with congested blood vessels at the serosal surface; however, no diverticulum, stricture, or tumors were found. Lymph nodes sampled showed only reactive hyperplasia. The patient had an uncomplicated postoperative recovery.

The case presented here is unique in that the most common risk factors for a phytobezoar were absent. In retrospect, key pieces of history should have raised sufficient concern for phytobezoar to be included on the initial differential diagnosis. This patient had a virgin abdomen and good dentition, thus eliminating the most common predisposing factors for phytobezoars prior surgery and poor dentition. The patient did, however, present with several less common risk factors for phytobezoar formation. For example, he had a lifelong history of postprandial abdominal symptoms which was not elicited during presurgical interviews. Since childhood he felt a bloating sensation and abdominal pain approximately two to three hours after eating. It was accompanied by a “squirting” sensation in his umbilical region and a pain that felt like a needle sticking him. Between the ages of 14 and 18 he was instructed by his pediatrician to take 2 teaspoons of Metamucil per day which seemed to relieve his symptoms. This may be consistent with subclinical congenital narrowing of the terminal ileum, which is supported by pathology findings of a narrow ileal lumen of the removed specimen, as shown in Figure 4. He also endorsed a history heartburn and acid reflux though to what extent is difficult to determine. Though he denied the use of a proton pump inhibitor, he reported frequent use of Maalox, Pepto Bismol, and Tums. This likely decreased the acidity level of the stomach. Four months prior to admission he ended a 4year history of heroin use which may have contributed to gastrointestinal immotility; however, there is only one reported case of a bezoar associated directly with opiate use to the authors' knowledge [36,37]. Though recently diagnosed with diabetes, it was likely longstanding due to the fact that his presentation at the time of diagnosis was diabetic retinopathy associated with a high HgbA1C level. Therefore, it is likely that diabetes was also a contributing factor toward gastric immotility in this patient. He recently increased his intake of fruits and vegetables in an effort “to eat healthier,” which included 2/3 of a can of mixed vegetables the day prior to admission (accounting for the corn, string beans, and carrots found in his small bowel) and pineapples two to three times per week. Unfortunately, this patient's good intentions likely contributed to the development of a phytobezoar at this point in time.

In conclusion, phytobezoars, while rare, should be suspected in cases involving several minor predisposing factors of phytobezoar formation, such as diabetes mellitus, opiate abuse, and seemingly harmless changes in diet that increase vegetable fiber intake. A long history of gastrointestinal problems, regardless of severity, should increase clinical suspicion of phytobezoars. CT findings of a mottled-gas, heterogenous transition point in the context of an acute abdomen should raise clinical suspicion of phytobezoars even if previous surgery and poor dentition are absent from the patient's history. It should also prompt a more detailed history including factors listed in Table 1.

## Figures and Tables

**Table 1:** Gastrointestinal Dysfunctions Associated with Bezoars.

Category	Subcategory	Example	Reference
I. Mechanical	IA. pyloric dysfunction or elimination	gastroileostomy, gastrojejunostomy	[3]
	IB. gastroparesis	diabetes mellitus, autonomic neuropathy, hypothyroidism, mixed connective tissue disease	[2,16]
	IC. narrowing and compaction	strictures from prior surgery or Crohn's, radiationinduced stenosis, tumor	[3,17,21,22]
	ID. dilataion and collection	congenital diverticulum	[19,20]
	IE. gastronintestinal immotility	hypothyroidism, opiates [only 1 case]	[2,16,36]
II. Chemical	IIA. hypoacidity	vagotomy, chronic antacid use	[3]
	IIB. increased intake of indigestible matter	poor dentition/mastication, increased intake of vegetable fiber, hair	[4,5]
	IIC. peculiar interaction between ingested matter and gastrointestinal environment	unripened persimmons, inspissated milk	[2]
